# Spondyloarthropathies in Autoimmune Diseases and Vice Versa

**DOI:** 10.1155/2012/736384

**Published:** 2012-02-15

**Authors:** Oscar M. Pérez-Fernández, Rubén D. Mantilla, Paola Cruz-Tapias, Alberto Rodriguez-Rodriguez, Adriana Rojas-Villarraga, Juan-Manuel Anaya

**Affiliations:** ^1^Center for Autoimmune Diseases Research (CREA), School of Medicine and Health Sciences, Universidad del Rosario, Carrera 24 # 63-C-69, Bogotá, Colombia; ^2^Rheumatology Unit, Riesgo de Fractura-CAYRE IPS, Bogotá, Colombia

## Abstract

Polyautoimmunity is one of the major clinical characteristics of autoimmune diseases (ADs). The aim of this study was to investigate the prevalence of ADs in spondyloarthropathies (SpAs) and vice versa. This was a two-phase cross-sectional study. First, we examined the presence of ADs in a cohort of patients with SpAs (*N* = 148). Second, we searched for the presence of SpAs in a well-defined group of patients with ADs (*N* = 1077) including rheumatoid arthritis (RA), systemic lupus erythematosus (SLE), and Sjögren's syndrome (SS). Among patients with SpAs, ankylosing spondylitis was observed in the majority of them (55.6%). There were two patients presenting with SS in the SpA group (1.4%) and 5 patients with autoimmune thyroiditis (3.5%). The global prevalence of ADs in SpAs was 4.86%. In the ADs group, there were 5 patients with SpAs (0.46%). Our results suggest a lack of association between SpAs and ADs. Accordingly, SpAs might correspond more to autoinflammatory diseases rather than to ADs.

## 1. Introduction

Spondyloarthropathies (SpAs) are a group of interrelated diseases with joint inflammatory involvement such as arthritis (axial and peripheral) and extraarticular involvement such as uveitis, enthesitis, psoriasis, and inflammatory bowel disease (IBD). This group of diseases is characterized by familial aggregation, absence of rheumatoid factor, and association with human leukocyte antigen (HLA)-B27 [1]. Classically, SpAs have been classified as ankylosing spondylitis (AS), Reiter syndrome (RS), reactive arthritis (ReA), psoriatic arthritis (PsA), IBD-associated SpA, and forms called undifferentiated SpA (uSpA) that do not meet the criteria for previous categories [2]. However, currently, there is a new classification for SpAs. This new classification includes two types of SpAs: axial and peripheral SpA depending on the predominant spinal or peripheral involvement [3, 4] and extraarticular involvement such as anterior uveitis or IBD, which are also considered part of the SpA group [5].

Autoimmune diseases (ADs), in turn, are a clinical syndrome caused by the loss of immune tolerance and characterized by T- or B-cell activation leading to tissue damage in the absence of any other evident cause [6]. Criteria for AD definition have been described and revisited [7]. These criteria, which include direct and indirect proof as well as circumstantial evidence [6], are described in Table 1. However, in many diseases labeled as autoimmune, there are several limitations to fulfill the concept of autoimmunity, which are mainly related to the lack of direct proof (autoantibodies and cell-mediated immunity). Conversely, autoinflammation, defined as self-directed tissue inflammation, is characterized by activation of the innate immune system determined by local factors at specific disease-prone sites [8]. Since polyautoimmunity (i.e., the presence of two or more well-defined ADs in a single patient) is one of the major clinical characteristics of ADs, our purpose was to look for the association between SpAs and ADs. To do so, a cross-sectional two-phase study was undertaken. First, the presence of ADs in a cohort of patients with SpAs was examined. Second, we searched for the presence of SpAs in a well-defined group of patients with ADs including rheumatoid arthritis (RA), systemic lupus erythematosus (SLE), and Sjögren's syndrome (SS).

## 2. Materials and Methods

### 2.1. Study Population

A total population of 1,077 patients from our RA, SLE, and SS database was reviewed. This database consists of 671 patients with confirmed RA, 239 with confirmed SLE, and 167 with confirmed SS. All patients are followed at the Center for Autoimmune Diseases Research (CREA) in Bogota, Colombia. All patients met four or more of the American College of Rheumatology (ACR) criteria for classification of RA and SLE [9, 10]. All patients with SS satisfied 4 or more of the diagnostic criteria for primary SS proposed by the European Community Study Group [11]. All of them required objective salivary gland involvement (i.e., focus score >1).

 A cohort of 148 patients with SpAs was consecutively evaluated and their clinical records reviewed. All SpAs patients were classified according to accepted international criteria for each disease. AS patients met modified New York criteria [12] and PsA patients satisfied CASPAR criteria [13]. For ulcerative colitis (UC) diagnosis was made on the basis of clinical suspicion supported by appropriate macroscopic findings on sigmoidoscopy or colonoscopy, typical histological findings on biopsy, and negative stool examinations for infectious agents. For Crohn's disease (CD) the diagnosis depended on demonstrating focal, asymmetric, and often granulomatous inflammation. However, the studies selected varied according to the presenting manifestations, physical findings, and complications [14]. IBD-associated SpA diagnosis required criteria for any type of SpA plus findings of UC or CD as was explained. For ReA, diagnosis was done based on the European Spondylarthropathy Study Group (ESSG) preliminary criteria for the classification of SpAs [15] and taking into account the fact that an antecedent of previous infection was required. Patients that did not meet the criteria for any SpA but satisfied the criteria for SpAs were classified as uSpA. Patients were also classified on the basis of the Assessment of SpondyloArthritis International Society (ASAS) criteria for axial and peripheral involvement [3, 4]. Patients with only extraarticular manifestations were classified as extraarticular SpA [5]. Patients with previous diagnostic of hypothyroidism were evaluated for autoimmune thyroiditis (AT) by searching of both antithyroglobulin (anti-Tg) and antithyroperoxidase antibodies (anti-TPO).

### 2.2. Clinical Variables

Information on patient demographics and cumulative clinical and laboratory manifestations over the course of the disease was obtained either by verification during discussion with the patient, an expert evaluation by a rheumatologist, or by chart review and were recorded in a standard and validated data-collection form for that purpose. A search was done for data on patients with any type of SpA and concomitant diagnoses of RA, SLE, and SS. Each patient's diagnosis was confirmed by review of clinical records using the criteria listed above (RA, SLE, SS, and SpAs).

 The institutional review board at the School of Medicine and Health Sciences of the Universidad del Rosario approved the study design, and all the patients signed the informed consent form.

### 2.3. Laboratory Tests

HLA-B27 was determined by flow cytometry or DNA typing. Antinuclear antibodies were determined by indirect immunofluorescence on HEp-2 cells. Rheumatoid factor was measured by turbidimetry. The detection of the specific antibodies, such as anti citrullinated cyclic peptide (anti-CCP), native anti-DNA, anti-RNP, anti-Sm, anti-Ro, anti-Tg, and anti-TPO antibodies were measured by Enzyme-Linked ImmunoSorbent Assay (ELISA) by using commercial kits (QUANTA Lite ELISAs, INOVA Diagnostics, Inc. San Diego, CA, USA) following the manufacturer's instructions.

### 2.4. Statistical Analysis

Univariate analysis was done on SpA patients. For the normal variables, mean and standard deviations (SD) are reported, and, for the nonnormal variables, interquartile ranges (IQR) are reported. Kolmogorov-Smirnov or Shapiro-Wilk tests were done to evaluate normality as appropriate. Differences between variables were analyzed by using T student, *X* ^2^, Fisher's exact test, ANOVA test, or Kruskall-Wallis as appropriate. Groups presenting *n* ≤ 5 were excluded from analysis. Bonferroni test was done when significant differences were found in ANOVA. In all the cases, a *P* value < 0.05 was considered as significant. Data were managed using the Statistical Package for the Social Sciences software (SPSS v18 for Windows).

## 3. Results

### 3.1. General Characteristics of Two Groups of Study

A total of 148 patients with SpAs were included. AS was observed in 55.6%, PsA in 21.5%, uSpA in 16.7%, IBD-associated SpA in 4.2%, and ReA in 2.1% of the patients evaluated. Disease was predominant in males for all subtypes, except for uSpA, where a higher prevalence of females as compared with males was observed, 66.7% versus 33.3%, respectively.  Table 2 summarizes the main clinical findings observed in the patients with SpAs at any time during the course of the disease, and Table 3 shows laboratory characteristics in patients with SpA and hypothyroidism.

Regarding the group of ADs, age at onset was significant lower in SLE patients than RA and SS patients. Otherwise, mean age of patients was higher in patients with RA than in patients with SS and SLE. As expected, all the ADs in this study were more frequent in women (Table 4).

### 3.2. ADs in Patients with SpAs

There were two patients presenting with SS in the SpA group (1.4%), one with AS, and other with PsA. There were no patients with RA or SLE observed in the SpA group; however, one patient with uSpA presented with HLA-B27, rheumatoid factor, and anti-CCP antibodies but did not fulfill the classification criteria for RA.

Hypothyroidism was present in 14 patients (9.5%). All of them were women. Of these patients, five (3.5%) met criteria for AT. Three were observed in the AS group, one in the PsA group and one in the uSpA group. Global prevalence of ADs in the SpA group was 4.86%. According to the new classification of SpAs, the presence of ADs was similar in both axial and peripheral SpA (Table 5).

### 3.3. SpAs in Patients with ADs

There was no patient with concomitant AS in the group of patients with RA or in that of patients with SLE (Table 6). Only one case of AS (0.59%) was found among 167 patients with SS. This patient did not belong to the same group of SpAs. Among all patients with ADs analyzed, the prevalence of AS was <1%. With respect to PsA, one case was found in the RA group (0.15%) and none in SLE or SS groups. The prevalence of PsA in all patients analyzed was also low (<1%) just as in the case of RA. IBD was presented in two patients (0.3%) in the RA group and in one patient (0.41%) in SLE group. Prevalence of IBD in the groups of ADs was extremely low (<1%). The overall prevalence of SpAs was also low (0.46%).

### 3.4. Comparison between SpAs and ADs Prevalences

Prevalence of ADs in the general population is considered to be 3.23% [16]. No significant differences between the prevalence of ADs in SpAs and the prevalence of ADs in the general population were found in our study (*P* > 0.05).

The prevalence of SpAs in the general population is about 0.4% [17]. Significant differences between the prevalence of SpAs in ADs in our patients as compared to the prevalence of SpAs in the general population were not found (*P* > 0.05).

## 4. Discussion

The prevalence of SpAs in ADs observed in our study (0.46%) was similar to the prevalence described in the general population (<1%) [17–19]. For example, Haglund et al. [18] found a prevalence of 0.45% for SpAs in southern Sweden. In North America, the prevalence of SpAs has been reported to be 0.4% [17]. Other studies on Caucasians have shown that the frequency of AS ranges between 0.15% and 1.8% and for PsA between 0.02% and 0.2% [20].

 Sundquist et al. [21] analyzed the concordant and discordant associations between RA, SLE, and AS as well as the risk of siblings to develop these associations by using standardized incidence ratios (SIRs). They observed concordant association in siblings when AS was compared with AS (SIRs = 17.14). In contrast, AS was not associated with RA or SLE [21]. Information about the association of SpAs and RA is scarce, and few case reports have been published [22–24]. In 1981, one study including 184 patients with AS or RS showed that five of them had concomitant diagnostic of RA and two of these five patients presented also with Felty's syndrome [25]. In our study, no patient with coexisting RA and AS was observed. However, one patient with uSpA presented with anti-CCP antibodies, rheumatoid factor, and HLA-B27 but, at the time of the inclusion, the patient did not meet criteria for RA.

 There are reports of IBD in RA [26] and SLE [27]. The present study reports a prevalence of 0.28% for IBD in all ADs, 0.3% in RA patients, and 0.41% in SLE. No patient with IBD was observed in SS. One study on North Americans reported elevated risk for RA in patients with IBD, showing an odds ratio (OR) of 2.7 with 95% confidence intervals (95% CI) between 2.4 to 3.0. However, the same study indicated a higher risk for AS (OR: 7.8; 95% CI: 5.6–10.8) than for RA [28]. Another study including 37 patients with IBD showed only one patient with peripheral arthritis and positive anti-CCP antibodies [29].

Concerning SLE, coexistence of AS is very rare, and this association has been suggested to occur in patients who carry one or two susceptibility alleles for both diseases [30]. Furthermore, shared environmental factors that remain to be identified may also contribute to triggering both diseases [30]. The recently published cases of the coexistence of SLE and AS corresponded to drug-induced SLE or lupus-like syndrome associated with anti TNF treatment in SpA patients [31–33]. In our study, we did not observe patients with SpAs in the SLE group and vice versa.

 There are few reports about the coexistence of SS and AS. Kobak et al. found SS in 10% of patients with AS in Turkey [34]. Brandt et al. reported a prevalence of 7.6% for SS in 105 patients with SpAs in Germany [35]. In our study, the prevalence of SS was 1.4% in all the patients with SpA. Different results could be related to diagnostic strategies (i.e., active search by performing autoantibodies and minor salivary gland biopsy systematically), ethnicity, and geography (i.e., environmental factors). We have no additional evidence of SpAs in other series stressing the scarce information in this regard.

 Hypothyroidism (of any cause) was observed in 9.5% of SpAs, and all were women. This prevalence is significantly higher than the prevalence of hypothyroidism in the general population, which is considered to be 1%-2% [36] (*P* < 0.001). Although these results might indicate that SpAs patients have an increased risk of hypothyroidism, the design of this study was not intended to answer this question. Therefore, further research in this topic is required. Of the 14 patients presenting with hypothyroidism, 5 were diagnosed with AT. Thus, the prevalence of AT in SpAs in our study was 3.5%. According to the subtype of SpA, the prevalence of AT in AS, PsA, and uSpA was 3.8%, 3.2%, and 4.2%, respectively. One study carried out in Italian women with PsA showed a high prevalence of AT as compared with controls. In such study, 28% of PsA had anti-Tg and 14% anti-TPO antibodies [37]. In our study, we observed a lower prevalence of AT in PsA (Table 5), which could be due to differences in gender, sample size, ethnicity, geography, and searching strategies.

 A case of AS and multiple sclerosis (MS) has been reported [38]; however, no patients with MS were observed in our cohort of SpAs.

 Concerning the clinical findings of our group of patients with SpAs, the results differ from other local studies to some degree. Londoño et al. [39] found a higher prevalence of uSpA (35.3%) than we did and a lower prevalence of PsA (9.4%) among their group of patients with SpAs. They observed a familial history of SpA in 18% of patients and did not find patients with IBD-associated SpA in contrast with our findings. Likewise, they observed male : female rate of 3 : 1, but the study was made in a military hospital, where male patients are more frequent. Another study done by Marquez et al. [40] in the city of Medellin, included 71 patients and showed similar results to ours, although a high prevalence of enthesitis (67%) was observed in their patients. These differences could be due to the heterogeneity of the Colombian population and limited number of patients analyzed in both studies as well as to ascertainment bias. Our patients come predominantly from the city of Bogota where the population admixture is higher than that in Medellin. One genetic study performed on Colombians with AS did not find significant differences in HLA-B27 subtypes between patients from Bogota (mestizos) and Cartagena (mulattos). However, clinical characteristics were not evaluated [41].

## 5. Conclusion

The findings presented in this study suggest a lack of association between SpAs and ADs. As a corollary, SpAs seem to be diseases that are not autoimmune in the strict sense even though they involve immunological reactions such as the overproduction of particular cytokines. Therefore, we consider them autoinflammatory diseases instead of autoimmune ones (Table 1) [8]. However, in spite of having included a large number of patients, our data were underpowered. Thus, to accurately investigate the association between SpAs and ADs, further multicenter and collaborative research is required, involving about 2000 cases and the same number of controls to allow a statistical power of 80% with a 5% of error.

## Figures and Tables

**Table 1:** Classification criteria for autoimmune diseases in humans. Comparison between ADs and autoinflammatory diseases.

			Diseases					
			SLE	RA	SS	AS	PsA	IBD
Direct proof	Antibody-mediated	Circulating ABs which alter the function	+	+	+			
		Localized ABs	+	+	+			
		IC located at lesion	+	+	+	+		
		Passive transference	+		+			
	Cells-mediated	In vitro T-cell proliferation in respond to autoantigen	+					
		In vitro T-cell transference to immune-deficient mice	+					
		In vitro T-cell cytotoxicity against target organ cells	+	+	+			

Indirect proof		Disease reproduction by experimental immunization	+	+	+	+	+	+
		Disease reproduction by idiotypes	+	+				+
		Spontaneous animal models	+	+	+	+	+	+
		Animal models produced by immune system deregulation	+	+	+	+	+	+

Circumstantial evidence		Auto-ABs	+	+	+			
		Other AD association	+	+	+		+	+
		HLA association	+	+	+	+	+	+
		Lymphocytic infiltration in target organ	+	+	+	+	+	
		Good response to immune suppression	+	+	+	+	+	+

AS: ankylosing spondylitis, PsA: psoriatic arthritis, IBD: inflammatory bowel disease, SS: Sjögren's syndrome, RA: rheumatoid arthritis, SLE: systemic lupus erythematosus, AD: autoimmune disease, ABs: antibodies, and IC: immune complexes.

**Table 2:** General characteristics of patients with SpAs.

	Classical classification [2]						New classification [3, 4]		
	All (*n* = 144)^a^	AS (*n* = 80)	PsA (*n* = 31)	uSpA (*n* = 24)	IBD-associated SpA (*n* = 6)	*P* value	Axial (*n* = 89)	Peripheral (*n* = 55)	*P* value
Mean (SD)									

Age, years	43.78 (11.08)	42.23 (10.33)	50.81 (13.00)	41.28 (8.45)	38.96 (6.02)	*P* < 0.05^b, c^	41.80 (10.15)	46.54 (11.84)	*P* = 0.018
Age at onset, years	34.77 (11.39)	33.24 (10.71)	41.23 (11.20)	33.71 (11.52)	29.33 (6.77)	*P* < 0.05^b^	33.05 (10.42)	37.62 (12.35)	*P* = 0.017
Age at diagnostic, years	39.24 (10.98)	37.58 (10.27)	45.65 (12.43)	37.54 (9.31)	34.17 (7.83)	*P* < 0.05^b, c^	37.40 (10.03)	42.00 (11.95)	*P* = 0.017

Characteristic, *n* (%)									

Percentage	100	55.6	21.5	16.7	4.2	NA	60.13	37.16	NA
Male^d^	82 (56.9)	45 (56.3)	20 (64.5)	13 (54.2)	2 (33.3)	NS	51 (57.39)	31 (56.4)	NS
Female	62 (43.1)	35 (43.8)	11 (35.5)	11 (45.8)	4 (66.7)	NS	38 (42.7)	24 (43.6)	NS
Low back pain	111 (77.1)	80 (100)	10 (32.3)	15 (62.5)	4 (66.7)	*P* < 0.001^b, c, e^	89 (100)	50 (90.9)	*P* < 0.001
Peripheral arthritis	73 (50.7)	22 (27.5)	24 (77.4)	22 (91.7)	3 (50)	*P* < 0.001^b, c^	23 (25.8)	50 (90.9)	*P* < 0.001
Enthesitis (heel)	26 (18.1)	18 (22.5)	2 (6.5)	4 (16.7)	2 (33.3)	NS	20 (22.5)	6 (10.9)	NS
Enthesitis (other sites)	34 (23.6)	19 (23.8)	6 (16.1)	8 (33.3)	2 (33.3)	NS	22 (24.7)	12 (21.8)	NS
Dactylitis	28 (19.4)	10 (12.5)	9 (29)	8 (33.3)	1 (16.7)	NS	11 (12.4)	17 (30.9)	*P* = 0.006
Psoriasis	28 (19.4)	0	28 (90.3)	0	0	*P* < 0.001^b, f, g^	2 (2.2)	26 (47.3)	*P* < 0.001
IBD	6 (4.2)	0	0	0	6 (100)	*P* < 0.001^e, g, h^	2 (2.2)	4 (7.3)	NS

HLA-B27									

Positive	43/86 (50)	27/54 (50)	2/11 (18.2)	14/17 (82.35)	0	NA	29/59 (49.15)	14/27 (51.85)	NA
Negative	43/86 (50)	27/54 (50)	9/11 (81.8)	3/17 (17.65)	2/2 (100)	NA	30/59 (50.85)	13/27 (48.15)	NA
No data available	58/144 (40.3)	26/80 (32.5)	20/31 (64.5)	7/24 (29.16)	4/6 (66.66)	NA	30/89 (33.7)	28/55 (50.9)	NA

AS: ankylosing spondylitis, PsA: psoriatic arthritis, uSpA: undifferentiated spondyloarthropatty, IBD: inflammatory bowel disease, ReA: reactive arthritis, AD: autoimmune disease, HLA: human leukocyte antigen, NA: not applicable, and NS: non significant.

^a^Neither ReA (*n* = 3) nor exclusive extraarticular SpA were included.

^b^Significant differences between AS and PsA.

^c^Significant differences between AS and uSpA.

^d^Significant differences in gender for all categories.

^e^Significant differences between AS and IBD-associated SpA.

^f^Significant differences between PsA and uSpA.

^g^Significant differences between PsA and IBD-associated SpA.

^h^Significant differences between uSpA and IBD-associated SpA.

**Table 3:** Hypothyroidism in SpAs.

	Classical classification [2]					New classification [3, 4]		
	All (*n* = 144)^a^	AS (*n* = 80)	PsA (*n* = 31)	uSpA (*n* = 24)	*P* value	Axial (*n* = 89)	Peripheral (*n* = 55)	*P* value
Hypothyroidism (%)	14/148 (9.5)	8/80 (10)	4 (12.9)	2 (8.3)	NS	9 (10.1)	5 (9.1)	NS
Anti-TPO positive	5/14 (35.7)	3/8 (37.5)	1/4 (25)	1/2 (50)	NS	3/9 (33.33)	2/5 (40)	NS
Anti-Tg positive	1/14 (7.14)	1/8 (12.5)	0	0	NS	1/9 (11.11)	0	NS
Both anti-TPO and anti-Tg positives	1/14 (7.14)	1/8 (12.5)	0	0	NS	1/9 (11.11)	0	NS

AS: ankylosing spondylitis, PsA: psoriatic arthritis, uSpA: undifferentiated spondyloarthropatty, NS: nonsignificant, anti-TPO: anti-thyroperoxidase, and anti-Tg: antithyroglobulin.

^a^Neither ReA (*n* = 3) nor exclusive extraarticular SpA were included.

**Table 4:** Age and gender of patients with ADs and SpAs.

Characteristic	RA (*n* = 671)	SLE (*n* = 239)	SS (*n* = 167)	SpAs (*n* = 148)	*P* value
Age, mean (SD)	51.8 (12.34)	37.1 (14.63)	50.5 (13.91)	43.78 (11.08)	<0.001^†^
Age at onset, mean (SD)	38.7 (13.47)	29.03 (13.02)	44.2 (13.72)	34.77 (11.39)	<0.001^¶^
Male (%)	18.3	18.2	5.3	56.9	
Female (%)	81.7*	81.8*	94.7*	43.1	

SS: Sjögren's syndrome, RA: rheumatoid arthritis, SLE: systemic lupus erythematosus, SpAs: spondyloarthropathies, and SD: standard deviation.

^†^Significant differences were observed between the following groups: RA versus SLE, RA versus SpAs, SLE versus SS, SLE versus SpAs, and SS versus SpAs.

^¶^Significant differences were observed between the following groups: RA versus SLE, RA versus SS, RA versus SpAs, SLE versus SS, SLE versus SpAs, and SS versus SpAs.

*Females were more prevalent in each group (*P* < 0.001), but not in the SpAs group.

**Table 5:** ADs in patients with SpAs.

	Classical classification [2]				New classification [3, 4]		
	All (*n* = 144)^a^	AS (*n* = 80)	PsA (*n* = 31)	uSpA (*n* = 24)	Axial (*n* = 89)	Peripheral (*n* = 55)	*P* value
SS	2 (1.39)	1 (1.3)	1 (3.22)	0	1 (1.12)	1 (1.81)	NS
RA	0*	0	0	0*	0	0*	NA
SLE	0	0	0	0	0	0	NA
AT	5 (3.47)	3 (3.75)	1 (3.22)	1 (4.16)	3 (3.37)	2 (3.63)	NS

Total	7 (4.86)	4 (5)	2 (6.45)	1 (4.16)	4 (4.5)	3 (5.45)	NS

AS: ankylosing spondylitis, PsA: psoriatic arthritis, uSpA: undifferentiated spondyloarthropatty, SS: Sjögren's syndrome, RA: rheumatoid arthritis, SLE: systemic lupus erythematosus, AT: autoimmune thyroiditis, AD: autoimmune disease, SpAs: spondyloarthropathies, NA: not applicable, and NS: nonsignificant.

^a^Neither ReA (*n* = 3) nor exclusive extraarticular SpA were included.

*One patient presented with monoarthritis, HLA-B27, anti-CCP, and rheumatoid factor.

**Table 6:** Prevalence of SpAs in patients with ADs.

*n* (%)	RA (*n* = 671)	SLE (*n* = 239)	SS (*n* = 167)	All (*n* = 1077)
AS	0	0	1 (0.59)	1 (0.092)
PsA	1 (0.15)	0	0	1 (0.092)
IBD	2 (0.3)	1 (0.41)	0	3 (0.28)

All SpAs	3 (0.44)	1 (0.41)	1 (0.59)	5 (0.46)

SLE: systemic lupus erythematosus, RA: rheumatoid arthritis, SS: Sjögren's syndrome, AS: ankylosing spondylitis, PsA: psoriatic arthritis, IBD: inflammatory bowel disease, and SpAs: spondyloarthropathies.
